# The novel myxofibrosarcoma cell line MUG-Myx1 expresses a tumourigenic stem-like cell population with high aldehyde dehydrogenase 1 activity

**DOI:** 10.1186/1471-2407-13-563

**Published:** 2013-12-01

**Authors:** Birgit Lohberger, Nicole Stuendl, Elisabeth Wolf, Bernadette Liegl-Atzwanger, Andreas Leithner, Beate Rinner

**Affiliations:** 1 Department of Orthopaedic Surgery, Medical University of Graz, Auenbruggerplatz 5, A-8036, Graz, Austria; 2 Department of Paediatric Orthopaedics, Medical University Graz, Graz, Austria; 3 Institute of Pathology, Medical University of Graz, Graz, Austria; 4 Center for Medical Research, Medical University of Graz, Graz, Austria

**Keywords:** ALDH1, Cancer stem cells, Myxofibrosarcoma, SNP analysis, STR profiling

## Abstract

**Background:**

Myxofibrosarcoma comprises a spectrum of malignant neoplasms withprominent myxoid stromata, cellular pleomorphism, and distinct curvilinear vascular patterns. These neoplasms mainly affect patients in the sixth to eighth decades of life and the overall 5-year survival rate is 60–70%.

**Methods:**

After the establishment of the novel myxofibrosarcoma cell lines MUG-Myx1, cells were characterized using short tandem repeat (STR), copy number variation (CNV), and genotype/loss-of-heterozygosity (LOH) analyses. The growth behaviour of the cells was analyzed with the xCELLigence system and an MTS assay. The tumourigenicity of MUG-Myx1 was proved in NOD/SCID mice. Additionally, a stem-like cell population with high enzymatic activity of aldehyde dehydrogenase 1 (ALDH1^high^) was isolated for the first time from myxofibrosarcoma cells using the Aldefluor® assay followed by FACS analysis.

**Results:**

The frozen primary parental tumour tissue and the MUG-Myx1 cell line showed the same STR profile at the markers D3S1358, TH01, D21S11, D18S51, Penta E, D5S818, D13S317, D7S820, D16S539, CSF1PO, Penta D, Amelogenin, D8S1179, TPOX, and FGY. Typically, myxofibrosarcoma gain and/or amplification was mapped to 7p21.3-q31.1, q31.1-q31.33, q33-q36.2, p21.3, p21.2, p14.1-q11.23, q31.33-q33, p21.2-p14.1, q11.23-q21.3, q36.2-q36.3, which, respectively are known to harbour tumour-associated genes, including TIF, BRAF, MLL3, SMO, and MET. Typically an LOH for myxofibrosarcoma on chr5 q21 was found. In addition, MUG-Myx1 ALDH1^high^ cells showed an upregulation of the ABC transporter ABCB1 and ABCG2; higher c-Myc, E-cadherin and SOX-2 expression; and a higher potential for tumourigenicity and proliferation levels.

**Conclusion:**

The new myxofibrosarcoma cell line MUG-Myx1 was established to enrich the bank of publicly available cell lines, with respect to providing comprehensive genetic and epigenetic characterization. Furthermore, because of their tumourigenicity, the cell line is also suitable for in vivo experiments.

## Background

Myxofibrosarcoma is the most common sarcoma in elderly patients and is characterized histologically by a multinodular growth pattern and variably prominent myxoid stroma. The tumour is mainly composed of spindle cells with variable cytologic atypia accentuated along curvilinear vessels [1]. Clinically, increasing grades and stages of the tumors are frequently seen in myxofibrosarcomas after relentless local recurrences, which may eventually lead to metastatic diseases [1-3]. Recurrence has been shown to occur in spite of repeated surgery involving wide local excisions and negative surgical margins [2]. Furthermore, metastatic myxofibrosarcomas are often refractory to current treatment strategies and constitute the primary cause of sarcoma-related death [1,3,4].

Permanent cell lines derived from primary sarcomas offer the opportunity to study functional alterations in sarcoma biology. The new myxofibrosarcoma cell line MUG-Myx1 was established to enrich the bank of publicly available cell lines, allowing comprehensive genetic and epigenetic characterization. Furthermore, because of their tumourigenicity, the cell line is also suitable for in vivo experiments. To develop novel prognostic adjuncts and therapeutic interventions, it is of paramount importance to elucidate the molecular determinants correlated with tumour aggressiveness and metastatic spread in myxofibrosarcoma progression. Important factors in potential therapeutic benefits are cancer stem cells (CSCs), which are defined as cells within a tumour that possess the capacity to renew themselves and generate the heterogeneous lineages of cancer cells that comprise the tumour [5,6]. Ginestier et al. showed that aldehyde dehydrogenase 1 (ALDH1) is a marker of normal and malignant human mammary stem cells and a predictor of a poor clinical outcome for breast cancer patients [7]. High ALDH1 activity characterises stem cell populations in many cancer types including human multiple myeloma, pancreatic cancer, breast cancer, and soft tissue sarcomas [8-10].

The present study describes the clinical, morphologic, and cytogenetic features of the newly established myxofibrosarcoma cell line, MUG-Myx1. An Aldefluor® assay and fluorescence-activated cell sorting (FACS) analysis were used to isolate stem-like ALDH1^high^ cells and ALDH1^low^ cells. Furthermore, we analysed the two subpopulations for their cell proliferation properties, expression of stem cell markers and ABC transporters, and tumourigenicity.

## Methods

### Patient history

A 66-year-old Caucasian man presented himself at the Department of Orthopaedic Surgery, at the Medical University of Graz, Austria, in April 2010 after an intra-lesional resection of a myxofibrosarcoma G3 on the left ventral thorax conducted at an outside institution. Radiography and magnetic resonance imaging (MRI) revealed postoperative haemato-seroma. Computer tomography of the thorax, abdomen and pelvis revealed no further lesions. In the same month, a wide resection was performed at our department and the thorax was reconstructed with a prolene net. A postoperative histopathological evaluation revealed a myxofibrosarcoma G3 with the resection margins free of disease. Postoperative chemotherapy with Epirubicine and Iphosphamide was performed and, in addition, radiotherapy was recommended. However, the patient refused this treatment. The research reported in this study was conducted adhering to the highest principles of human welfare according to the Consort declaration on clinical research design and the Helsinki declaration on medical protocols and ethics. The study protocol and the informed consent of the patients were approved by the ethics committee of the Medical University Graz (vote #20-430ex08/09; valid until 25.09.2013). The patient was extensively informed and gave his written approval.

### Cell culture procedures

The tumour tissue was obtained immediately after surgical removal. After mechanical disaggregation of the tumour tissue into 1–2 mm^3^ pieces, the minced tissue was enzymatically digested with 2 mg/ml collagenase B (Roche Diagnostics, Mannheim, Germany) for approximately 20 hours under constant rotation at 37°C. Cells were then centrifuged at 1400 rpm for 5 min and washed twice with PBS. Collected cells were plated in Dulbecco’s-modified Eagle’s medium (DMEM-F12; Invitrogen, Darmstadt, Germany), containing 10% foetal bovine serum (FBS; Invitrogen), 1% L-glutamine (Invitrogen), 100 units/ml penicillin (Invitrogen), 100 μg/ml streptomycin (Invitrogen) and 0.25 μg amphotericin B (PAA Laboratory, Pasching, Austria). Cells were kept at 37°C in a humidified atmosphere of 5% CO_2_ and passaged by trypsination upon reaching confluence. All cell cultures were periodically checked for mycoplasma by PCR.

### Immunohistochemical studies

#### Patient’s tumour

For the histopathological evaluation, the tumour was tested using the streptavidin-biotin peroxidase complex method with antibodies against Caldesmon (Dako, Glostrup, Denmark), S100 (Dako), CD34 (Neomarkers, Fremont, CA), Desmin, EMA, and Pan-CK (all Ventana Medical Systems, Tucson, AZ).

#### MUG-Myx1 characterization

For IHC analysis, cells were seeded at a concentration of 1 × 10^4^ cells on polystyrene culture slides (BD Biosciences, San Diego, US). When cell cultures reached approximately 70% confluence, slides were washed with PBS and fixed by exposure to formalin 4% for 10 minutes.

Cells were grown on culture slides (confluence 70–80%) and fixed with acetone for 10 min at -20°C. After drying and rehydration, the slides were treated with Large Volume UltraV-Block (ThermoScientific, Waltham, US) for 10 min at room temperature to block nonspecific binding, incubated with the primary monoclonal mouse anti-Vimentin antibody (Dako) for 30 min and, after several washing steps, incubated with the Cy2 conjugated sheep anti-mouse IgG secondary antibody (Jackson Immunoresearch, Suffolk, UK) at a dilution of 1:200 for 30 min. Nuclei were counterstained with DAPI (Invitrogen).

#### SCID mice tissue

IHC studies using the streptavidin-biotin peroxidase complex method were carried out on histological slides from ALDH1^high^ and ALDH1^low^ SCID mice tumours, employing an rabbit monoclonal primary antibody against the anti-Ki-67 (clone 30–9) (Ventana Medical Systems) using the BenchMark Ultra instrument (Ventana Medical Systems). Slides were photographed using an Olympus BX51 microscope with an Olympus DP71 microscope digital camera. The stained slides were scanned digitally and positive and negative cells were quantified using the ImageScope software (ImageScope Virtual Slide, version 6.25, Aperio Technol.,Vista, US). Positivity was determined by assessing the number of positive cells/number total cells.

### Cell proliferation analysis

#### MTS

1 × 10^3^, 5 × 10^3^, and 1 × 10^4^ MUG-Myx1 cells were seeded into 96-well microtiter plates (Brand, Voerde-Friedrichsfeld, Germany) and the CellTiter 96® AQ_ueous_ Assay (Promega, Mannheim, Germany) was performed after the manufacturer’s instructions at 24-, 48-, 72-, and 96-hour timepoints. The culture medium was used as a negative control.

#### xCELLigence system

The xCELLigence DP device from Roche Diagnostics (Mannheim, Germany) was used to monitor cell proliferation in real-time. Respectively 5 × 10^3^ and 1 × 10^4^ MUG-Myx1 cells were seeded in electronic microtiter plates (E-Plate™; Roche Diagnostic) and measured for 92 h with the xCELLigence system according to the instructions in the user’s manual. Cell density measurements were performed in quadruplicate with a programmed signal detection every 20 min. Data acquisition and analyses were performed with the RTCA software (version 1.2, Roche Diagnostics).

### Tumour formation in SCID mice

#### Tumourigenicity of MUG-Myx1

8 week old female/male NOD/SCID/IL-2rγnull (NSG-) mice (Charles River Laboratories, Sulzfeld, Germany) were xenotransplanted with the MUG-Myx1 cell line at passage 65. MUG-Myx1 (4 × 10^6^ cells) were suspended in 0.2 ml of serum-free medium and subcutaneously inoculated into the left flank of 10 mice. The mice were observed daily and the tumour growth was monitored. All animal work was done in accordance with a protocol approved by the institutional animal care and use committee at the Austrian Federal Ministry for Science and Research (BMWF) (vote 66.010/0160-II/3b/2012).

#### Tumourigenicity after cell sorting

Under the same conditions, eight mice were xenotransplanted. ALDH-stained MUG-Myx1 cells were separated by FACS analysis and cultured over two weeks. 1 × 10^6^ ALDH1^low^ cells were injected into the right flank, and 1 × 10^6^ ALDH1^high^ cells were injected into the left flank, of 8-week old female/male NOD/SCID/IL-2rγnull (NSG-) mice.

### Cell cycle analysis

5 × 10^5^ cells were fixed with 70% ice-cold ethanol for 10 min at 4°C. After washing, the cell pellet was re-suspended in PI-staining buffer (50 μl/ml PI, RNAse A, Beckman Coulter, US) and incubated for 15 min at 37°C. Cells were spiked with mononuclear cells (MNC) (positive diploid population control) then analysed by flow cytometry (FACSCalibur, BD Biosciences, San Jose, US). A minimum 10,000 events per sample were acquired and data were analysed by using CellQuest (BD Biosciences). The DNA index was calculated by calculating the geometric mean M2 (MUG-Myx1)/geometric mean M1 (MNCs).

### Cell line identification Power Plex® 16 system

Frozen tumour tissue was dissected into small pieces and re-suspended in 180 μl ATL buffer (Qiagen, Hilden, Germany). Cell pellets (3.5 × 10^5^) from MUG-Myx1 (p2 and p43) were re-suspended in 200 μl PBS; subsequently 20 μl Proteinase K and 200 μl AL Buffer (Qiagen) were added. DNA preparations were performed using the QIAamp DNA Mini kit (Qiagen) in accordance with the manufacturer’s protocol. After normalizing the DNA, 1 μl of each sample was amplified using the Power Plex® 16 System (Promega, Vienna, Austria) in a 10 μl reaction. One μl of the product was mixed with Hi-Di formamide (Applied Biosystems Inc., Foster City, US) and Internal Lane Standard (ILS600), denatured and fractionated on an ABI 3730 Genetic Analyzer (Applied Biosystems Inc.). The resulting data were processed and evaluated using ABI Genemapper 4.0 (Applied Biosystems Inc.).

### Affymetrix SNP 6.0 array processing and analysis

Genomic DNA was isolated from MUG-Myx1 cells using the QIAmp DNA Kit (Qiagen). The Affymetrix GeneChip Human Mapping SNP 6.0 array was performed as described in the Genome-Wide Human SNP Nsp/Sty 6.0 User Guide (Affymetrix Inc., Santa Clara, US). SNP 6.0 data were imported and normalized using the Genotyping Console 4.0 program default settings. All samples passing QC criteria were subsequently genotyped using the Birdseed (v2) algorithm. We used 60 raw HapMap data generated with the Affymetrix Genome-Wide Human SNP Array 6.0 as a reference. Data were obtained from the Affymetrix website and used for normalization. For the visualization of the copy number state and LOH, the Chromosome Analysis Suite 1.1 software (Affymetrix Inc.) was used.

### Aldefluor® assay and separation of the ALDH1^high^ cell population by FACS analysis

Aldehyde dehydrogenase (ALDH) enzyme activity in viable cells was determined using a fluorogenic dye-based Aldefluor® assay (Stem Cell Technologies, Grenoble, France) according to the manufacturer’s instructions. 1 × 10^6^/ml cells were suspended in Aldefluor® assay buffer containing ALDH substrate (Bodipy-Aminoacetaldehyde) and incubated for 45 min at 37°C. As a reference control, the cells were suspended in buffer containing Aldefluor® substrate in the presence of diethylaminobenzaldehyde (DEAB), a specific ALDH1 enzyme inhibitor. The brightly fluorescent ALDH1-expressing cells (ALDH1^high^) were detected in the green fluorescence channel (520–540 nm) of the FACSAria (BD Biosciences) and the data were analysed using FACS DIVA software (BD Biosciences).

### Reverse transcription quantitative real-time -PCR (RT-qPCR)

RT-qPCR was performed in order to determine the relative expression of the ABC transporter genes ABCG2/BCRP1 and ABCB1/MDR1, and the stemness markers SOX-2, c-Myc, and E-cadherin. Total RNA was isolated with RNeasy Mini Kit (Qiagen) according to the manufacturer’s recommended protocol. RNA quality was analysed using the Agilent RNA 6000 Nano Kit and the Bioanalyzer 2100 (Agilent Technologies, Santa Clara, CA). All RIN values were between 9.8 and 10.0. DNA was digested with 1 U DNase (Fermentas, St. Leon-Rot, Germany) per μg RNA. One μg RNA was reverse transcripted using RevertAid cDNA Synthesis Kit (Fermentas). RT-qPCR reactions were performed in triplicate using the Platinum SYBR Green Super Mix with ROX (Invitrogen) on AB7900HT (Applied Biosystems Inc.). The reference genes glyceraldehyde 3-phosphate dehydrogenase (GAPDH), β-actin (ACTB) and hypoxanthine phosphoribosyltransferase (hprt-n) were used for normalization and to demonstrate their stable expression in different tissues [11]. The following primers were used for RT-qPCR: QuantiTect primer assays (Qiagen) for ABCB1 (ID QT00081928), ABCG2 (ID QT00073206), c-Myc (ID QT00062069), SOX-2 (ID QT00237601), and E-cadherin (ID QT00080143). The expression level (CT) of the target gene was normalized to the reference genes (GAPDH, ACTB, and hprt-n) (ΔCt) and then the ΔCt of the test sample was normalized to the ΔCt of the controls (ΔΔCt). Finally, the expression ratio was calculated with the 2-ΔΔCt –method [12].

### Statistical analysis

The outcome variables were expressed as mean ± SD. The student’s unpaired *t*-test and the exact Wilcoxon’s test were used to evaluate differences between groups with the PASW statistics 18 software (IBM Corporation, Somers, NY). Two-tailed *P*-values below 0.05 were considered statistically significant. Graphic data were prepared with SigmaPlot® (Systat Software Inc., San Jose, US).

## Results

### Establishment of the novel myxofibrosarcoma cell line MUG-Myx1

**Figure 1:**
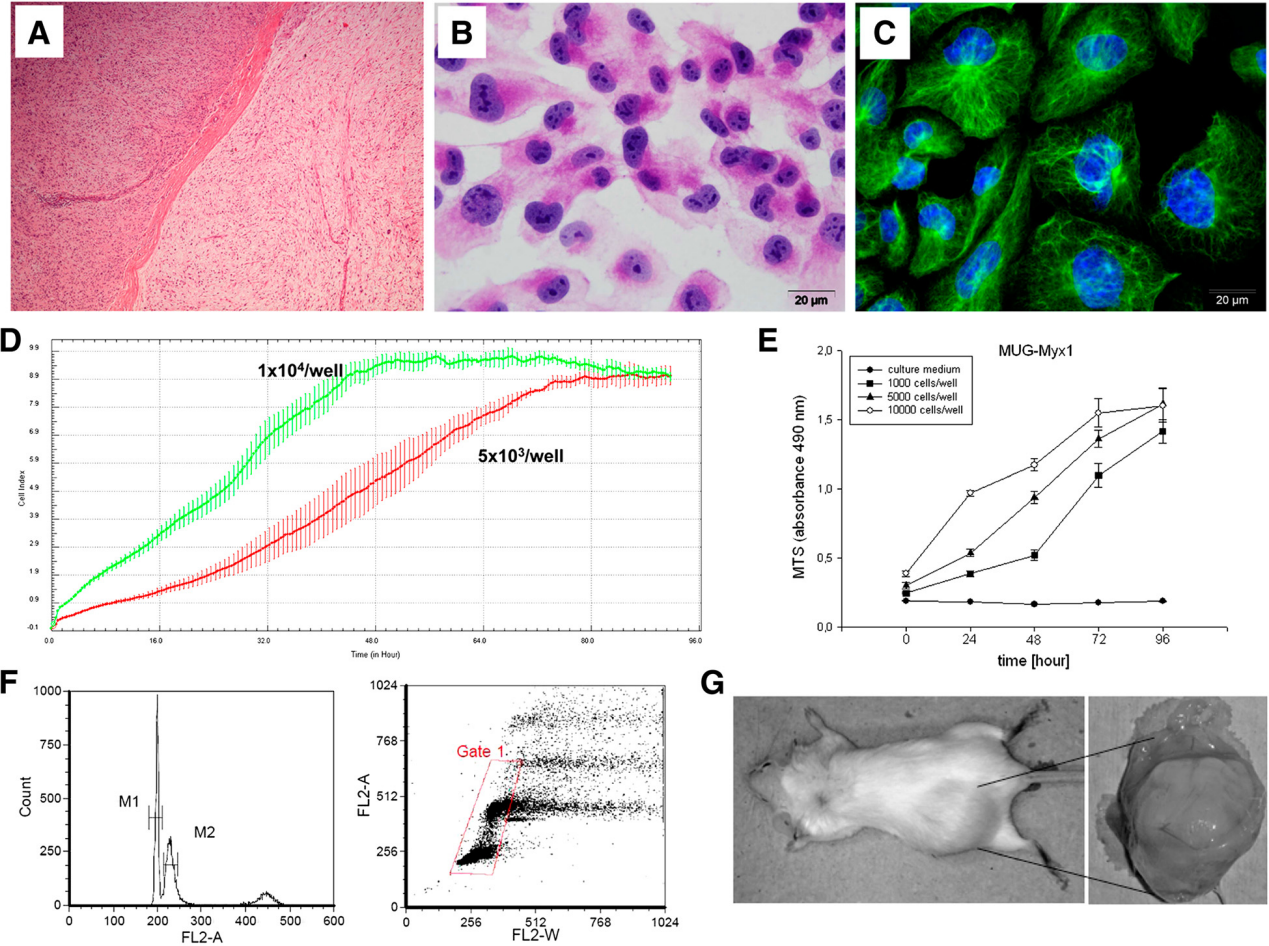
**Establishment and characterization of the myxofibrosarcoma cell line MUG-Myx1. (A)** IHC analysis on patient tumour tissue showed poorly differentiated tumour components (left) in connection with better differentiated tumour areas with myxoid stroma and curvilinear blood vessels (right). **(B)** The hematoxylin and eosin (H&E) staining of the MUG-Myx1 cell line showed spindle and multinucleated tumour cells. **(C)** Strong expression of Vimentin of the MUG-Myx1 cell line confirmed the mesenchymal origin of the tumour cells; nuclei were stained with DAPI. **(D)** Dynamic proliferation curves for MUG-Myx1; 5 × 10^3^ and 1 × 10^4^ cells were seeded per well and measured with the xCELLigence system. **(E)** MTS proliferation analysis revealed a 24 h doubling time. **(F)** The DNA index of 1.15 indicated hyperdiploid tumour cells (left). Gating strategy of cell cycle analysis, to exclude doublets from the total population (right). **(G)** MUG-Myx1 Tumour formation in NOD/SCID/IL-2rγnull (NSG-) mice.

Haematoxylin and eosin (HE)-stained slides of the above-mentioned patient revealed a myxofibrosarcoma G3. The tumour was composed of tumour areas showing a myxoid stroma with classic curvilinear tumour vessels, as well as areas showing a high grade tumour component. Immunohistochemical (IHC) analysis of the patient’s tumour revealed only focal SMA positivity, whereas tests for Desmin, Caldesmon, S100, CD34, EMA, and Pan-CK were negative (Figure 1A).

After crushing and enzymatically digesting the tumour tissue, the cells were successfully grown. During the course of cultivation, the cells were regularly cryopreserved. Cells grew to be adherent as a monolayer. The cells were passaged more than 100 times and were in culture for 12 months, however, the morphology of MUG-Myx1 cells did not change significantly during long-term cultivation. HE staining showed cells with prominent nucleoli and abundant cytoplasm (Figure 1B). The mesenchymal origin of the tumour was confirmed by high vimentin expression (Figure 1C).

In order to elicit the growth behaviour of the cells, they were detected in triplicate with the xCELLigence System (Roche Applied Science). Using the RTCA 1.2.1 software (ACEA Biosciences Inc., San Diego, US), the population doubling time of the MUG-Myx1 cells was calculated at 24 h at 37°C in a humidified atmosphere (Figure 1D). Additionally, the growth behaviour of three different cell counts was investigated with the MTS assay after 24–96 hours (Figure 1E).

To characterize the MUG-Myx1 cell line, the following analyses were carried out: definition of the ploidy status, tumourigenicity in NOD/SCID mice, short tandem repeat (STR) analysis, copy number variation (CNV), and genotype/loss-of-heterozygosity (LOH) analysis. The DNA index was calculated by analysing the geometric mean M2 (MUG-Myx1) 229.0/geometric mean M1 (MNCs) 199.0. This results in DNA index of 1.15, which means the cells were hyperdiploid (Figure 1F). MUG-Myx1 (p65) successfully formed tumours in 8 of 10 transplanted mice. The take rate was very fast; small nodules were palpable 2 weeks after inoculation, and the tumours grew to 1.2–2.3 cm in diameter 5 weeks later. The remaining two mice died. One representative mouse and its accompanying tumour is shown in Figure 1G (n = 8). The success rate of MUG-Myx1 cells growing in NOD/SCID/IL-2rγnull (NSG-) mice was 80%. For the identification of the cell line, we used the Power Plex® 16 System. The frozen primary parental tumour tissue and the MUG-Myx1 cell line (p2 and p43) showed the same STR profile at the markers D3S1358, TH01, D21S11, D18S51, Penta E, D5S818, D13S317, D7S820, D16S539, CSF1PO, Penta D, Amelogenin, D8S1179, TPOX and FGY. All values are summarized in Table 1.

**Table 1:** STR genotype of primary tumour, a low passage (p2) and a high passage (p43) MUG-Myx1 cell line

	**Tumour tissue**	**p2**	**p43**
**D3S1358**	17,18	17,18	17,18
**TH01**	6,8	6,8	6,8
**D21S11**	26,31	26,31	26,31
**D18S51**	17,24	24	17,24
**Penta E**	12,17	12,17	12,17
**D5S818**	13	13	13
**D13S317**	9,12	12	9,12
**D7S820**	10,11	10,11	10,11
**D16S539**	12,13	12	12,13
**CSF1PO**	11	11	11
**Penta D**	9,12	9,12	9,12
**AMEL**	XY	XY	XY
**vWA**	15,19	15,19	15,19
**D8S1179**	13	13	13
**TPOX**	8	8	8
**FGA**	18,20	18,20	18,20

### Cytogenetic findings

#### Chromosomal copy number analysis

**Figure 2:**
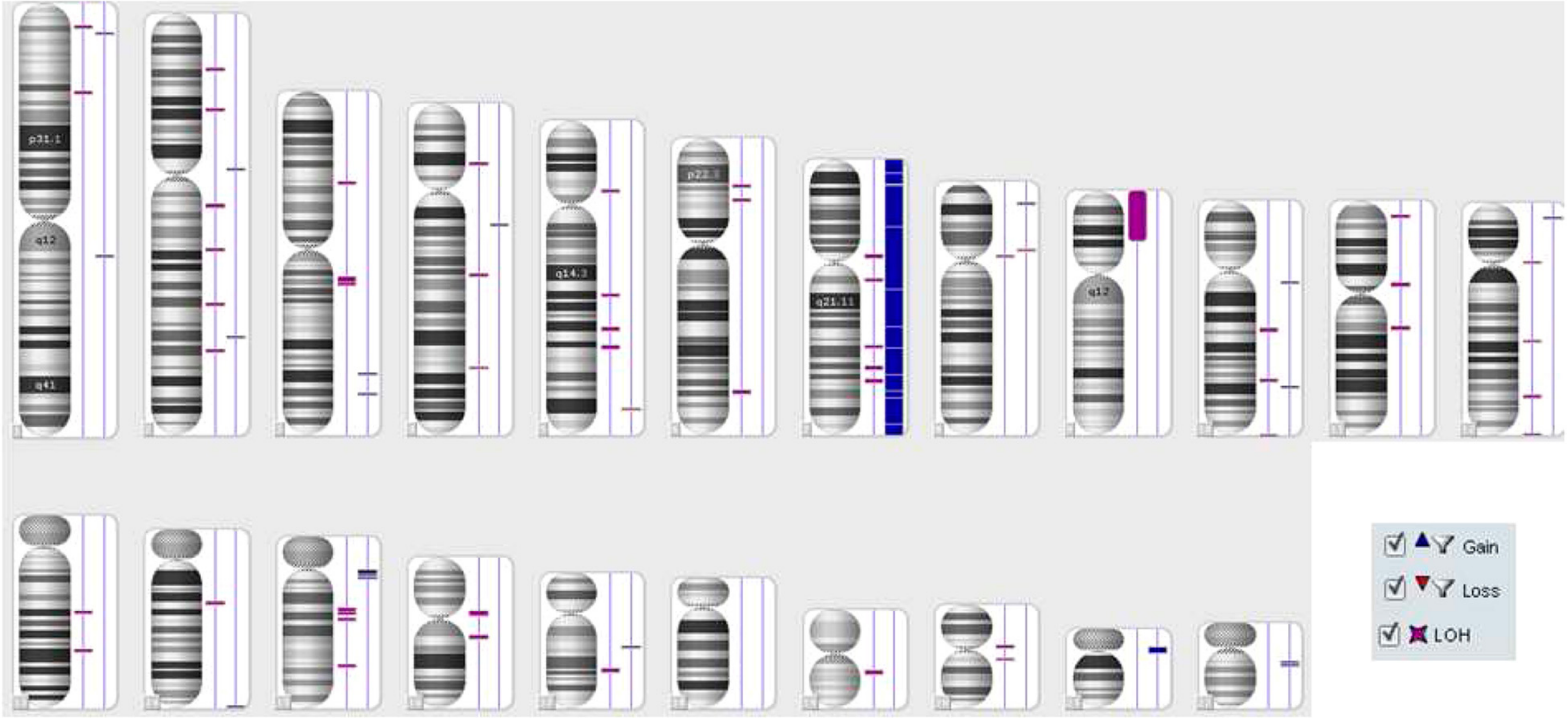
**Cytogenetic findings.** Overview of chromosomal CNVs and LOHs observed for the passage 43 of the MUG-Myx1 cell line. The first line represents LOHs (violet mark), the second line represents gains (blue mark) and losses (red mark).

A CNV and LOH analysis of the cell line reveals gains, losses and copy neutral LOHs (uniparental disomy), as are summarized in Figure 2 and Tables 2 and 3. Typically myxofibrosarcoma gain and/or amplification were mapped to 7p21.3-q31.1, q31.1-q31.33, q33-q36.2, p21.3, p21.2, p14.1-q11.23, q31.33-q33, p21.2-p14.1, q11.23-q21.3, q36.2-q36.3. These loci are respectively known to harbour tumour-associated genes, including TIF, BRAF, MLL3, SMO, and MET. However, losses tended to be small changes, which mapped only to chr5 q34 and chr8 p11.22, and acquired uniparental disomy (aUPD), also known as copy number neutral LOH, occurs prominently in the cell line (Figure 2). Typical LOH for myxofibrosarcoma on chr5 q21 were found.

**Table 2:** Gain and Loss at chromosomal regions and localised cancer genes in the myxofibrosarcoma cell line MUG-Myx1

**CN state**	**Type**	**Chromosome**	**Cytoband start**	**Cytoband end**	**Associated cancer genes**
3	Gain	1	p36.13	p36.13	
3	Gain	1	q21.1	q21.1	PDE4DIP
3	Gain	2	q32.1	q32.1	
4	Gain	2	p11.2	p11.2	
3	Gain	3	q26.31	q26.31	
4	Gain	3	q26.1	q26.1	
4	Gain	4	q13.2	q13.2	
1	Loss	5	q34	q34	
3	Gain	7	p22.3	p21.3	CARD11;PMS2
3	Gain	7	q21.3	q31.1	
3	Gain	7	q33	q36.2	TRIM24;KIAA1549;BRAF;EZH2;KMT2C
3	Gain	7	p21.3	p21.2	ETV1
3	Gain	7	p21.2	p21.2	
3	Gain	7	p14.1	q11.23	IKZF1;EGFR;SBDS;ELN;HIP1
3	Gain	7	q31.33	q33	SMO
3	Gain	7	p21.2	p14.1	HNRNPA2B1;HOXA9;HOXA11;HOXA13;JAZF1
3	Gain	7	q33	q33	CREB3L2
3	Gain	7	q11.23	q21.3	AKAP9;CDK6
3	Gain	7	q31.1	q31.33	MET
3	Gain	7	q36.2	q36.3	MNX1
3	Gain	8	p23.1	p23.1	
1	Loss	8	p11.22	p11.22	
3	Gain	10	q11.22	q11.22	
4	Gain	10	q25.1	q25.1	
3	Gain	10	q11.22	q11.22	
3	Gain	12	p13.31	p13.31	
3	Gain	14	q32.33	q32.33	
3	Gain	15	q11.2	q11.2	
3	Gain	15	q11.2	q11.2	
4	Gain	15	q11.1	q11.2	
3	Gain	17	q21.31	q21.31	
3	Gain	21	p11.1	q11.2	
3	Gain	22	q11.22	q11.22	
3	Gain	22	q11.23	q11.23	

**Table 3:** Loss of heterozygosity of the myxofibrosarcoma cell line MUG-Myx1

**Type**	**Chromosome**	**Cytoband start**	**Cytoband end**	**Associated cancer genes**
LOH	1	p36.22	p36.21	
LOH	1	p33	p32.3	CDKN2C
LOH	2	p23.1	p22.3	
LOH	2	q21.3	q22.1	
LOH	2	q24.3	q24.3	
LOH	2	q32.3	q32.3	
LOH	2	p16.1	p16.1	
LOH	2	q12.3	q13	
LOH	3	q13.13	q13.13	
LOH	3	q13.12	q13.13	
LOH	3	p21.2	p21.1	BAP1
LOH	4	q22.3	q23	RAP1GDS1
LOH	4	p15.1	p15.1	
LOH	4	q31.3	q31.3	
LOH	5	p13.1	p13.1	
LOH	5	q23.1	q23.1	
LOH	5	q23.3	q31.1	ACSL6
LOH	5	q21.1	q21.1	
LOH	6	q24.3	q24.3	
LOH	6	p22.2	p22.1	
LOH	6	p21.31	p21.31	FANCE
LOH	7	p11.2	p11.2	EGFR
LOH	7	q32.1	q32.1	
LOH	7	q11.22	q11.22	
LOH	7	q31.1	q31.1	
LOH	7	q31.31	q31.31	
LOH	8	p11.21	p11.1	HOOK3
LOH	9	p24.3	p21.1	JAK2;CD274;CD273;NFIB;PSIP2;MLLT3;CDKN2a(p14)
LOH	10	q22.1	q22.2	
LOH	10	q26.3	q26.3	
LOH	10	q24.32	q24.32	NFKB2
LOH	11	p15.4	p15.4	
LOH	11	q13.4	q13.4	
LOH	11	p11.2	p11.12	
LOH	12	q24.33	q24.33	
LOH	12	p11.1	p11.1	
LOH	12	q21.2	q21.31	
LOH	12	q24.11	q24.13	ALDH2
LOH	13	q21.1	q21.1	
LOH	13	q31.1	q31.1	
LOH	14	q21.1	q21.2	
LOH	15	q15.1	q15.3	
LOH	15	q21.1	q21.1	
LOH	15	q24.3	q24.3	
LOH	15	q21.1	q21.2	
LOH	16	q11.2	q12.1	
LOH	16	p11.2	p11.2	
LOH	17	q22	q23.2	CLTC
LOH	19	q13.12	q13.13	
LOH	20	q11.22	q11.22	
LOH	20	p11.21	p11.1	

### MUG-Myx1 cell line displays a considerable fraction of ALDH1^high^ stem-like cells

**Figure 3:**
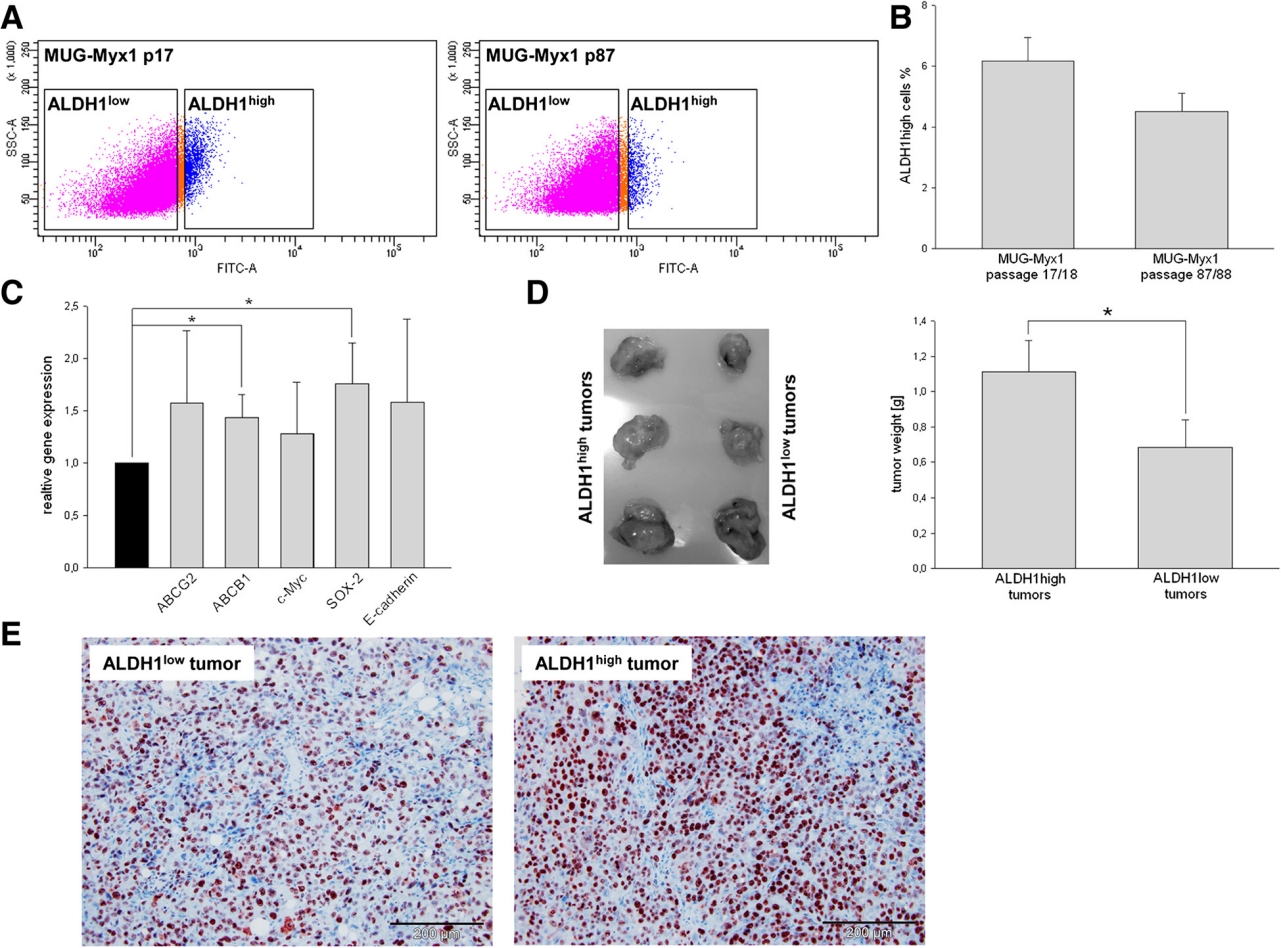
**Characterization of the ALDH1 **^**high **^**subpopulation. (A)** Aldehyde dehydrogenase 1 (ALDH1) expression in MUG-Myx1 cells using the Aldefluor® assay. **(B)** ALDH1 expression in percentage of gated cells. **(C)** The normalized expression levels from ABC transporter genes and stemness markers in ALDH1^high^ cells compared to ALDH1^low^ control cells (black bar). **(D)** SCID mice ALDH1^low^ and ALDH1^high^ tumours differed significantly in their tumour weights. **(E)** The IHC analysis using anti-Ki-67 proliferation marker revealed an increased proliferation level of ALDH1^high^ as compared to ALDH1^low^ mouse tumours.

We used the Aldefluor® assay followed by FACS analysis to assess the presence and quantity of ALDH1^high^ cell populations in the MUG-Myx1 cell line. In order to set a marker for ALDH1^high^ cells, diethylaminobenzaldehyde (DEAB) control cells were used to ensure the accuracy of the analysis. MUG-Myx1 cells in a low passage (p17) and in a high passage (p87) (Figure 3A) were treated in the presence of the ALDH1 inhibitor DEAB or stained with Aldefluor® reagent, which are defined as ALDH1^low^ and ALDH1^high^ cells. Sorting experiments were performed a minimum of seven times on each passage. The amount of ALDH1^high^ cells given on average ± SD was 6.16 ± 1.75% for the lower passage (n = 7) and 4.53 ± 1.55% for the higher passage of MUG-Myx1 (n = 8) (Figure 3B).

### The mRNA expression of ABC transporter and stemness marker are upregulated in MUG-Myx1 ALDH1^high^ cells

The relative expression of two major drug transporters ABCG2/BCRP1 and ABCB1/MDR1 were determined by RT-qPCR (n = 7). The ALDH1^high^ population of MUG-Myx1 demonstrated, with statistical significance, an increased expression level of ABCB1 (ratio 1.43 ± 0.22; p = 0.0046) compared to ALDH1^low^ control cells (ratio = 1), whereas the increase of ABCG2 was not significant (ratio 1.57 ± 0.69) (Figure 3C). Furthermore, we investigated whether ALDH1^high^ cells are enriched for expression of genes that have been postulated to play key roles in stem cell biology, such as c-Myc, E-cadherin, and SOX-2. Quantitative RT-PCR showed a significantly increased expression of SOX-2 in the ALDH1^high^ population (ratio 1.75 ± 0.39; p = 0.0051). Similarly, a slight but not significant increase in the expression of c-Myc (ratio 1.28 ± 0.49) and E-cadherin (ratio 1.58 ± 0.79) in the ALDH1^high^ fraction was observed (n = 7) (Figure 3C).

### MUG-Myx1 ALDH1A1^positive^ cells show higher tumourigenicity and proliferation

After separation by FACS Aria (BD Bioschiences), MUG-Myx1 cells derived from the 87^th^ passage successfully formed transplanted tumours in all eight transplanted mice. After five weeks, the ALDH1^high^ cells formed significantly larger tumours with the same cell amount and same latency period as in the mice injected with ALDH1^low^ cells. Furthermore, they differed significantly in their tumour weights (0.68 ± 0.41 g vs 1.11 ± 0.47 g; p = 0.012; n = 8) (Figure 3D). Successful engraftment was determined by pathological examination of the formalin-fixed, paraffin-embedded (FFPE) material of the tumour samples. High mitotic rate and high proliferative index were confirmed by IHC with the proliferation marker Ki-67. Using the ImageScope software, Ki-67 tissue samples ALDH1^low^ and ALDH1^high^ tumour slides were quantified after IHC staining. ALDH1^high^ tumours from all eight mice displayed an increased proliferation level as compared to ALDH1^low^ tumours (Ki-67 positivity: 0.206 ± 0.039 vs 0.067 ± 0.041; p = 1.11E-07; n = 8). A representative staining of one pair of tumours is shown and summarized in Figure 3E.

## Discussion

Myxofibrosarcoma is a malignant neoplasm with variably prominent myxoid stroma, cellular pleomorphism, and a distinct curvilinear vascular pattern and represents the most common sarcoma in elderly patients, with a slight predominance in males [1]. Local, often repeated recurrences, unrelated to histological grade, occur in up to 50–60% of cases [2,13-15].

To develop novel therapeutic interventions, it is highly desirable to establish new human primary cell lines to elucidate the molecular determinants correlated with tumour invasion and metastatic spread. The established MUG-Myx1 cell line can be maintained in long-term cultures with a 24-hour doubling time. The parent tumour and the cultured tumour cells clearly demonstrated the typical morphological and histological features of myxofibrosarcoma. In order to characterize our MUG-Myx1 cell line, we have determined the DNA ploidy status. Aneuploidy is defined as an abnormal chromosome number that deviates from a multiple of the haploid as a consequence of gradual gains or losses of chromosomes in cancer cells that evolve into unstable complex karyotypes [16-18]. MUG-Myx1 cells revealed a DNA index of 1.15, which defined its hyperdiploid status. This result is in concordance with Huang et al., who found aneuploid or tetraploid DNA ploidy status in 75 well-characterized myxofibrosarcomas [4]. One key feature of cancer cells versus normal cells is chromosome instability, which is proposed to be critical for the initiation of tumourigenesis [19]. It was for this reason that we were interested in investigating the genomic integrity of MUG-Myx1 by SNP analysis. Gains in gene copy number drive the expression of oncogenes, whereas decreased gene dosage by hemizygous and/or homozygous deletion result in the inactivation of tumour suppressor genes [20]. Mertens et al. showed that the only recurrent gain involves chromosome 7, whereas losses primarily affect chromosomes 1, 3, 5, 6, 10, 12, 16, 17, and 19 [21]. There is mounting evidence that regional gains and/or high-level amplifications on chromosomal arm 7q are recurrently found in various types of bone and soft tissue sarcomas, including myxofibrosarcomas [22-26]. Interestingly, overexpression of the oncogene MET in myxofibrosarcoma, as a frequent event, was strongly related to higher grades and seems to have a causative function in conferring an aggressive phenotype [27]. Tsai et al. showed a strong correlation between CDK6 and MET gene copies on 7q in primary myxofibrosarcomas. CDK6 protein overexpression and gene amplification were both univariately associated with worse outcomes [28].

MUG-Myx1, especially, showed gains on chromosome 7, including at 7q31.1, the MET location, and can therefore represent an in vitro model for MET-target therapy investigations. Furthermore, Tuna et al. determined the frequency and distribution patterns of aUPD in soft tissue sarcoma and identified aUPD in myxofibrosarcoma at 1p35.1-p34.2 and 16q23.3-q24.1, which we were able to confirm in our newly established cell line [29]. Due to the fact that metastatic myxofibrosarcomas are often refractory to current treatment strategies and constitute the primary cause of sarcoma-related death [30,31], we also wanted to investigate stem-like cells in the MUG-Myx1 cell line. Based on the current CSC hypothesis, only a small subpopulation within the heterogeneous tumour population is capable of initiating and re-initiating tumours. The concept of CSCs was based on the observation that when cancer cells of many different types were assayed for their proliferative potential in various assays in vitro and in vivo, only a minority of cells showed extensive proliferation [32]. One widely accepted method for identifying CSCs is based on the enzymatic activity of ALDH1, a detoxifying enzyme responsible for the oxidation of intracellular aldehydes. The ground-breaking work of Ginestier et al. showed the potential applicability of quantifying ALDH activity in solid tumours [7]. In the future, ALDH activity could be used successfully as a CSC marker for many cancers, including soft tissue sarcomas [10,33-36]. The present results demonstrate that MUG-Myx1 cells contained a distinctive fraction of ALDH1^high^ cells, interestingly in a higher percentage in the lower passage (p17). Our group observed that the number of ALDH1^high^ decreased during the course of cultivation.

The higher expression of ABC transport proteins in stem cells as compared to non-stem cells results in a higher resistance of the stem cells to the toxic effects of chemotherapy drugs [37,38]. We analysed the mRNA expression of the two major drug transporters ABCG2/BCRP1 and ABCB1/MDR1. In the present study, both drug transporters were upregulated in MUG-Myx1 ALDH1^high^ cells. Thus, these genes may potentially be ideal targets for clinical cancer therapy. Because c-Myc has recently been recognized as an important regulator of stem cell biology, it may serve as a link connecting malignancy and “stemness” [39]. The introduction of c-Myc with other transcription factors (including SOX-2) generates the induction of pluripotent stem cells from differentiated cells [40]. Our quantitative RT-PCR data showed increased expression of c-Myc, SOX-2, and E-caherine in the ALDH1^high^ population. The ALDH1^high^ population showed a significantly higher tumour formation capacity and proliferation rate, consistent with the characteristics of the high ALDH1 activity phenotype in other cancer cells [41,42], which may indicate that ALDH1^high^ cells are partially responsible for tumour metastasis and recurrence and should be targeted during the cancer therapy.

## Conclusion

In conclusion, the well-characterized myxcofibrosarcoma cell line MUG-Myx1 will be a useful tool to gain further insights into the pathogenesis of myxofibrosarcoma and explore new treatment options. Targeting stem-like cells with increased ALDH1 expression may especially facilitate the development of better treatment for patients suffering from myxofibrosarcomas.

## Abbreviations

ALDH1: Aldehyde dehydrogenase 1; CNV: Copy number variation; CSC: Cancer stem cells; DEAB: Diethylaminobenzaldehyde; IHC: Immunohistochemical studies; FACS: Fluorescence-activated cell sorting; FFPE: Formalin-fixed, paraffin-embedded; HE: Haematoxylin and eosin; LOH: Loss-of-heterozygosity; STR: Short tandem repeat.

## Competing interests

The authors declare that they have no competing interests.

## Authors’ contributions

BL and RB conceived and supervised the study. NS, BL, and BR performed the experiments. BL, RB, BLA analyzed and interpreted the data. BL, RB, and BLA drafted and revised the manuscript. EW and AL provided technical support. All authors read and approved the final manuscript.

## Pre-publication history

The pre-publication history for this paper can be accessed here:

http://www.biomedcentral.com/1471-2407/13/563/prepub
